# Cytochalasin H Inhibits Angiogenesis *via* the Suppression of HIF-1α Protein Accumulation and VEGF Expression through PI3K/AKT/P70S6K and ERK1/2 Signaling Pathways in Non-Small Cell Lung Cancer Cells: Erratum

**DOI:** 10.7150/jca.85372

**Published:** 2023-04-28

**Authors:** Yuefan Ma, Zihan Xiu, Zhiyuan Zhou, Bingyu Huang, Jiao Liu, Xiaofeng Wu, Sanzhong Li, Xudong Tang

**Affiliations:** 1 Collaborative innovation center for antitumor active substance research and development, Guangdong Medical University, Zhanjiang 524023, P.R. China; 2 Institute of Biochemistry and Molecular Biology, Guangdong Medical University, Zhanjiang 524023, P.R. China; 3 Guangdong Key Laboratory for Research and Development of Natural Drugs, Guangdong Medical University, Zhanjiang 524023, P.R. China; 4 Guangdong Provincial Key Laboratory of Medical Molecular Diagnostics, Guangdong Medical University, Dongguan 523808, P.R. China.

In the original version of our article, “(Figure 2)” should be changed into “(Figure 3A)” in bottom line 7 of page 2002 (right). Moreover, there was an error in Figure 2F of the original version of our article. Specifically, the representative image of VEGF expression of H460 xenografted tumors in Figure 2F is incorrect. The correct image is provided below. This correction will not affect the results and conclusions. The authors apologize for any inconvenience this may have caused.

## Figures and Tables

**Figure 2:**
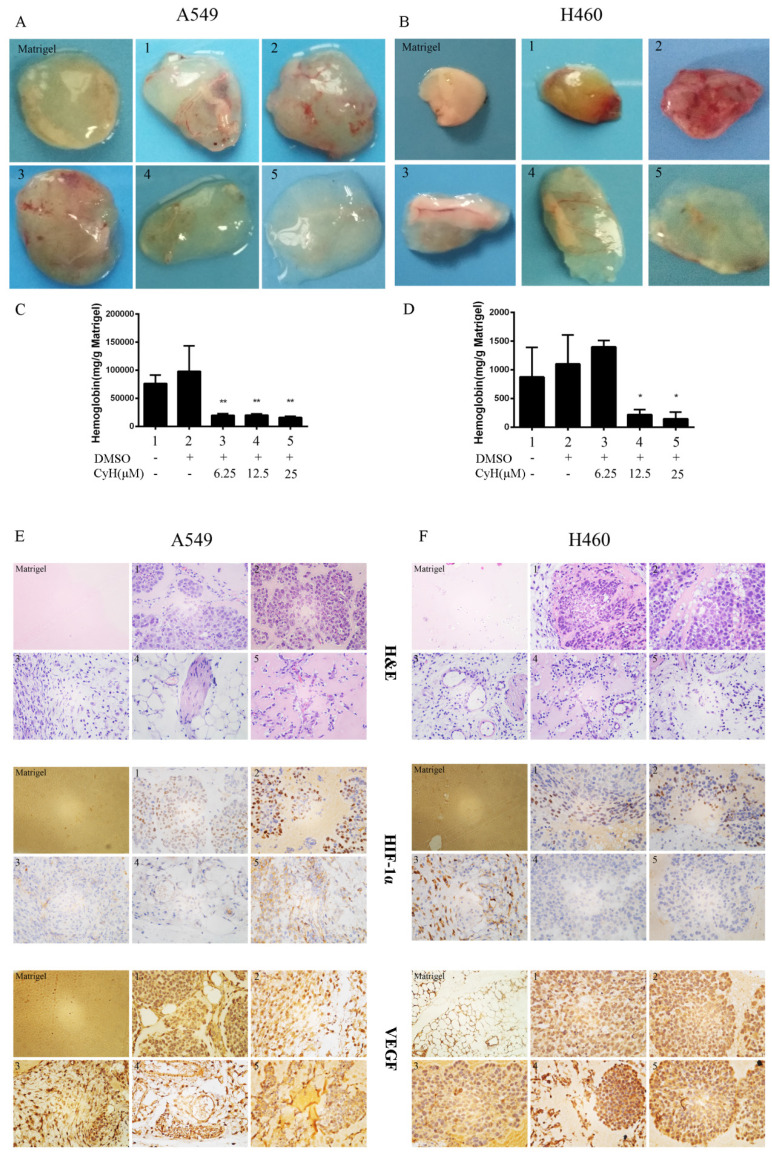
CyH inhibited angiogenesis *in vivo* and HIF-1α and VEGF protein expression in A549 and H460 NSCLC xenografts. A549 and H460 cells were treated with different concentrations (0, 6.25, 12.5, and 25 μM) of CyH. (A, B) Matrigel plugs. (C, D) The hemoglobin levels in Matrigel plugs. Hemoglobin content was expressed as (mg/g) of Matrigel plug. Immunohistochemical studies on the expression of HIF-1α and VEGF proteins in A549 (E) and H460 (F) xenografted tumors (original magnification, ×400). The results are representative of five independent experiments. Compared with control (lane 2), ^*^P<0.05, ^**^P<0.01.

